# Rubber seed oil and flaxseed oil supplementation on serum fatty acid profile, oxidation stability of serum and milk, and immune function of dairy cows

**DOI:** 10.5713/ajas.18.0573

**Published:** 2019-01-04

**Authors:** Yu Pi, Lu Ma, Hongrong Wang, Jiaqi Wang, Jianchu Xu, Dengpan Bu

**Affiliations:** 1 State Key Laboratory of Animal Nutrition, Institute of Animal Science, Chinese Academy of Agricultural Sciences, Beijing 100193, China; 2 College of Animal Science and Technology, Yangzhou University, Yangzhou 225009, China; 3 Kunming Institute of Botany, Chinese Academy of Sciences, Kunming 650201, China; 4 Hunan Co-Innovation Center of Safety Animal Production, Changsha, Hunan 410128, China; 5 CAAS-ICRAF Joint Laboratory on Agroforestry and Sustainable Animal Husbandry, Beijing 100193, China

**Keywords:** Vegetable Oils, Linolenic Acid, Polyunsaturated Fatty Acid, Lipid Oxidation, Immunity, Lactating Cows

## Abstract

**Objective:**

This study was designed to investigate the effect of diet supplementation with rubber seed oil and flaxseed oil on serum fatty acids profile, oxidation stability of serum and milk, and immune function of dairy cows.

**Methods:**

Forty-eight mid-lactation Holstein dairy cows were randomly assigned to one of four treatments for 8 wk, including basal diet (CON) or the basal diet supplemented with 4% rubber seed oil (RO), 4% flaxseed oil (FO) or 2% rubber seed oil plus 2% flaxseed oil (RFO) on a dry matter basis.

**Results:**

Compared with CON, all the oil groups increased the levels of *trans*-11 C18:1 (vaccenic acid), *cis*-9, *trans*-11 C18:2 (conjugated linoleic acid, CLA) and C18:3 (α-linolenic acid, ALA) in serum. Both the activity of glutathione peroxidase and catalase in serum and milk in oil groups were decreased, which were negatively correlated with the levels of *cis*-9, *trans*-11 CLA and ALA. The concentrations of proinflammatory factors (tumor necrosis factor α and interferon γ) in serum of oil groups were lower than that from the CON cows.

**Conclusion:**

These results indicate that diet supplementation with RO or FO could alter serum fatty acid profile and enhance the immune function of dairy cows. However, the negative effect on milk oxidation stability should be considered when feeding these n-3 polyunsaturated fatty acid-enriched oils in dairy production.

## INTRODUCTION

Vegetable oils, which are rich in polyunsaturated fatty acids (PUFA), have been used to alter the milk fatty acid (FA) composition, especially PUFA content, to improve milk fat quality [1,2]. The unsaturated fatty acids (UFA), especially the PUFA, are of vital importance in counteracting the occurrence of spontaneous lipid oxidation [3]. Studies reported that the susceptibility of lipid spontaneous oxidation increased with elevated concentrations of PUFA [4]. In addition, dietary sources high in n-3 PUFA could affect aspects of immune function such as lowering lymphocyte response to mitogens and natural killer cell ability [5]. Evidence also suggests that n-3 PUFA affects inflammation through altered gene expressions of inflammatory mediators [6]. Although n-3 PUFA may exhibit anti-inflammatory effects that contribute to alleviating of the severity of some autoimmune diseases, their inhibition on cell-mediated immunity, which could impact on host immune defenses, may be undesirable [7]. Furthermore, extensively consuming α-linolenic acid (ALA), one of the important n-3 PUFA, may suppress the immune function and health of dairy cows [8,9]. Thus, it is necessary to investigate the oxidative stability and immune function effects during the period of PUFA supplementation.

Rubber seed oil (RO) is a by-product of the rubber industry, and the main origin of RO being in Guangdong, Guangxi, and Yunnan provinces of China. In RO, the levels of total UFA and PUFA can amount to 83% and 59%, respectively [10]. Flaxseed oil (FO) and RO are both rich in ALA. In FO, ALA contributes approximately 55% of the oil’s total FA, whilst in RO the ALA content is lower at 22%. It is notable that previous rat toxicological and brine shrimp tests showed that RO had no acute toxicity effect and was not found to contain any hazardous linamarin [11]. Moreover, our previous study reported that RO and FO supplementation can increase milk production and alter milk fat FA composition by increasing the contents of ALA, vaccenic acid (VA) and conjugated linoleic acid (CLA) and decreasing the content of saturated fatty acid (SFA) [10]. However, whether diet supplementation with n-3 PUFA-enriched oils (RO and FO) impact on milk fat oxidation stability and cow’s health by affecting antioxidant capacity and immune function is still unknown. Thus, the objective of this study was to investigate the effect of RO and FO supplementation on blood FA composition, antioxidant capacity of serum and milk fat, and immune function of dairy cows.

## MATERIALS AND METHODS

The study was performed at Tianjin Mengde Dairy Farm (Tianjin, China). All procedures in this experiment were approved by the Institutional Animal Care and Use Committee of Chinese Academy of Agriculture Sciences (Beijing, China).

### Cows, experimental design and treatments

The details of animal experimental design were reported in a previous study [10]. Briefly, forty-eight mid-lactation healthy Chinese Holstein dairy cows (163±25.3 d in milk, 29.6±2.42 kg of milk/d, and parity 1.8±1.25) were randomly assigned to one of four treatments (n = 12) according to a completely randomized design. The total duration of the experiment was eight weeks, during which the cows were fed a basal diet (CON) or the basal diet supplemented with 4% RO, 4% FO, or 2% rubber seed oil plus 2% flaxseed oil (RFO) on a dry matter (DM) basis. The FO was supplied by Huajian Axunge Co., Ltd (Shanxi, China) and the RO was supplied by Kunming Institute of Botany, Chinese Academy of Sciences (Kunming, China). The ingredients and chemical composition of the diets are presented in Supplementary Table S1. The diet was formulated to meet or exceed the nutrient demand according to the Feeding Standards of Dairy Cattle, China NongYe HangYe Biaozhun/Tuijian-34 [12]. During the experimental period, cows were housed in a mechanically ventilated barn divided into 4 plots with 12 cows each and fed individually, with free access to fresh water. At the beginning of the study, cows were gradually adjusted to the experimental diets over a one-week period. Diets were fed as a total mixed ration (TMR) three times daily (05:30, 13:30, and 18:00 h) to ensure <10% refusals. The oils were stored at 4°C and were added fresh as the final component after mixing the other dietary ingredients. The TMR containing oil supplements were made once daily and stored under shade until fed at a later time. Cows were milked 3 times daily (at 05:00, 13:00, and 21:00 h) with individual milk yields recorded at each milking.

### Sampling, measurements, and analysis

The quantity of daily feed offered and refused was recorded for individual cows. Samples of TMR were collected daily and frozen at −20°C prior to subsequent analysis. Orts were sampled twice weekly from each cow, composited for each treatment, and frozen at −20°C for further analysis. Weekly representative samples of TMR from each treatment were analysed for DM content by oven-drying at 60°C to a constant weight and analysed for their FA profile according to the procedure of Sukhija and Palmquist [13].

Blood samples were collected at the end of week 7 of the experiment. Approximately 15 mL of duplicate blood samples from individual cows were collected from the coccygeal vein four hours after feeding [1]. Blood samples were collected into serum separator tubes (Serum Clot Activator, Greiner Bio-One GmbH, Kremsmünster, Austria), and centrifuged at 3,000×*g* for 15 min at 4°C to separate the serum. Duplicate milk samples from individual cows were collected on the last day of week 8. Milk samples were collected at 3 consecutive milkings and mixed based on the average milk production at each milking (morning, afternoon, and night; volume ratio: 4:3:3). All the samples were stored at −70°C for further analysis.

Serum FA analysis was conducted using the method ac cording to previous study [10]. The activities of superoxide dismutase (SOD), glutathione peroxidase (GSH-Px), catalase (CAT) and concentration of malondialdehyde (MDA) in serum and milk samples were determined using kits according to the manufacturer’s instructions (Nanjing Jiancheng Bioengineer Institute, China). Serum immunoglobulin (Ig) A, IgG, and IgM levels were determined by enzyme-linked immunosorbent assay (ELISA) with the cow IgA, IgG, and IgM ELISA kit commercially obtained from Bethyl Laboratories (Montgomery, TX, USA). Concentrations of interleukin (IL)-2, IL-4, IL-6, IL-10, interferon (IFN)-γ, tumour necrosis factor (TNF)-α, and prostaglandin E2 (PGE2) in the serum were measured with the cow ELISA kit (GSI Equined-Plasma/Serum DataSheet, Genorise Scientific, Inc., Glen Mills, PA, USA) following the manufacturer’s protocol.

### Statistical analysis

Data were analyzed as a completely randomized design using the MIXED procedure of SAS (version 9.2, SAS Institute Inc., Cary, NC, USA). The statistical model included cow as random effect, and treatment as fixed effects. For the statistical analysis of milk yield and FA intake, sampling time and sampling time×treatment were added to the model and analyzed using repeated measures. Orthogonal contrasts included: CON vs oil supplemented diets to test the effect of oil supplementation; RO vs FO to test the effect of high rubber seed oil vs high flaxseed oil intake; and RFO vs RO+FO to test the additive effect of rubber seed oil and flaxseed oil. The significance level was declared at p<0.05 and trends for significance were declared at p = 0.05 and a trend at p<0.10. Correlations between the FA intake, the levels of FA in serum and milk, the oxidation stability indexes in serum and milk, and the immune function indexes were assessed by Pearson’s correlation test using GraphPad Prism version 5.00 (GRAPHPAD Software, San Diego, CA, USA). Significant correlation was considered at p<0.05.

## RESULTS

### Fatty acid intake and milk production

**Figure 1:**
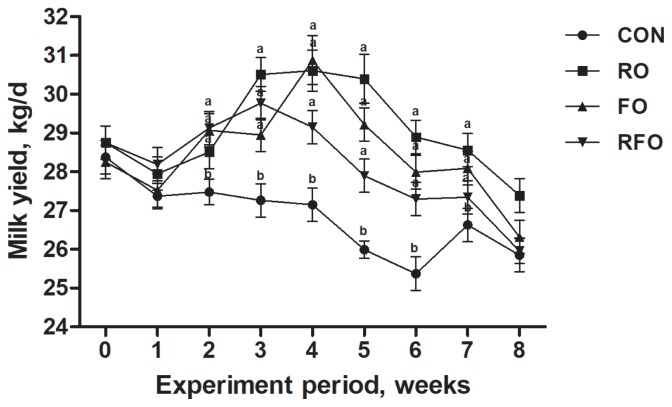
Average milk yield of treatment groups at week 0, 1, 2, 3, 4, 5, 6, 7, and 8. Control (CON) = diet without added oils; rubber seed oil (RO) = CON diet including rubber seed oil at 4% of dry matter (DM); flaxseed oil (FO) = CON diet including flaxseed oil at 4% of DM; rubber seed oil and flaxseed oil (RFO) = CON diet including rubber seed oil at 2% of DM plus flaxseed oil at 2% of DM. Data were presented as the mean±standard error of least squares means (n = 12). Means at the same week point with different letters (a, b) differ significantly (p<0.05) for treatment effect.

There was no difference in DMI and energy intake between the CON group and the oil groups, however, all the oil treatment groups showed increased intake of *cis*-9 C18:1, C18:2, ALA, total UFA, and total PUFA (p<0.05) (Table 1). Compared with FO group, RO group significantly decreased the amount of *cis*-9 C18:1, C18:2, ALA, total UFA, and total PUFA intake and increased the amount of SFA intake (p<0.05). In addition, oil treatment groups significantly increased milk yield compared with CON group (p<0.05) and there was no difference among the oil groups after week 1 (Figure 1).

### Fatty acid composition in serum and milk

The composition of FA in serum are shown in Table 2. Compared with CON group, oil groups significantly increased the levels of *trans*-11 C18:1 (VA), *cis*-9, *trans*-11 C18:2 CLA, ALA, and C20:5 (eicosapentaenoic acid, EPA) in serum, while markedly decreased the content of SFA. However, the concentrations of VA, *cis*-9, *trans*-11 CLA, ALA, and EPA in serum in FO groups were higher than that in RO (p<0.05). Feeding the blend of RO and FO (RFO) increased (p<0.05) the concentrations of VA and *cis*-9, *trans*-11 CLA in serum to a greater extent than feeding them separately. The composition of FA in milk were reported in our previous study [10]. Briefly, the data exhibited that the proportions of short-chain (C4:0, C6:0, C8:0, C10:0, C12:0, and C13:0) and medium-chain FAs (C14:0, C14:1, C15:0, C16:0, and C16:1) were lower (p<0.05) in milk from cows fed RO, FO, or RFO. The proportion of long-chain FAs (C18:0, *trans*-9 C18:1, VA, *cis*-9 C18:1, CLA, and ALA) in milk fat increased (p<0.05) in cows fed RO, FO, or RFO.

### Enzymatic radical scavenging systems in serum and milk fat

In serum, oil groups had no effect on the activity of SOD (p> 0.05) (Table 3). Both the concentrations of GSH-Px and CAT in cows receiving oils (RO, FO, or RFO) were lower than that in the CON (p<0.05). In addition, CSH-Px concentration in FO group was lower than that in RO group (p<0.05). The concentration of MDA in cows receiving FO were higher than that in cows receiving RO (p<0.05). Moreover, the concentration of MDA in the oil groups were increased compared with the CON group (p<0.10). In milk, the activity of SOD was decreased in oil treatment groups (RO, FO, or RFO) compared with the CON group (p<0.05). The concentration of CAT in cows receiving RO, FO, or RFO were lower than that in the CON (p<0.05). Moreover, the concentration of MDA in the oil groups were increased compared with the CON group (p<0.05).

### Antibody levels in serum

To investigate the effect of RO and FO feeding on systemic immune response in dairy cows, circulating IgA, IgG, and IgM antibody levels in serum were determined (Table 4). The concentration of IgG in cows receiving the CON markedly increased compared with that in the oil-supplemented treatments (p<0.05). Nevertheless, the concentration of IgG was higher (p<0.01) in RO group than that in FO group. In addition, the concentrations of IgA, IgG, and IgM in cows feeding the blend of RO and FO (RFO) had no difference (p<0.05) compared with that in cows feeding them separately.

### Prostaglandin E2 and cytokine concentrations in serum

As shown in Table 4, compared with the CON group, the concentrations of INF-γ and TNF-α in serum were decreased in oil groups (p<0.05). In addition, the concentrations of PGE2 (p<0.01), IL-4 (p<0.05), and IL-10 (p<0.10) in RO group were higher than that in FO group. Moreover, feeding the blend of RO and FO (RFO) resulted in lower concentrations of IL-4 than feeding them separately (p<0.10), whereas serum IL-4 level was not influenced (p>0.05) by dietary oils treatment. These results indicate diet supplementation with RO, FO, and RFO could decrease pro-inflammatory factors (INF-γ and TNF-α) in the serum of dairy cows, and cows fed with RO also showed an increase in the anti-inflammatory factors (IL-4 and IL-10) compared to cows offered FO.

### Correlation analysis

**Figure 2:**
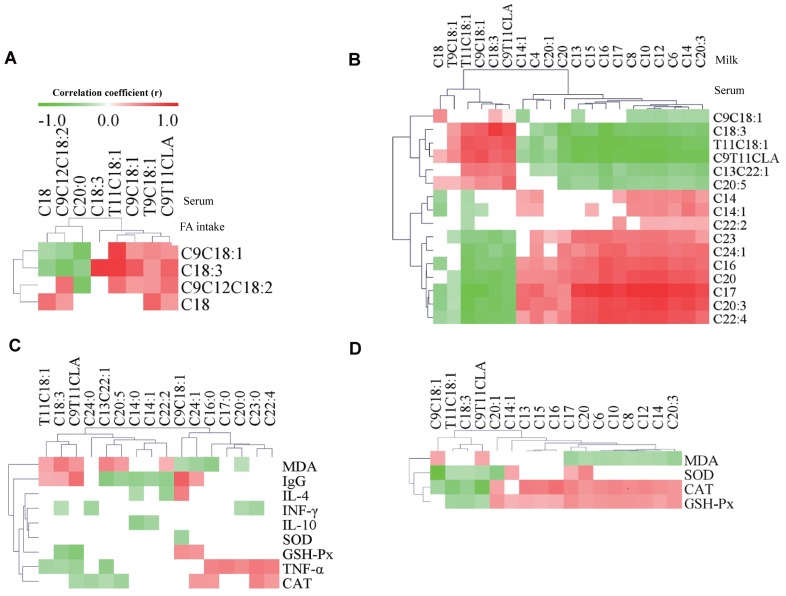
Correlation analysis between FA intake, the levels of FA in serum, the levels of FA in milk, the oxidation stability indexes in milk and serum, and the immune function indexes. (A) correlation between FA intake and the levels of FA in serum; (B) correlation between the levels of FA in serum and milk; (C) correlation between the levels of FA in serum and the oxidation stability and immune function indexes in serum; (D) correlation between the levels of FA in milk and the oxidation stability indexes of milk. The color is according to the Pearson correlation coefficient distribution; red represents significant positive correlation (p<0.05), green represents significantly negative correlation (p<0.05), white represents a non-significant correlation (p>0.05). CLA, conjugated linoleic acid; Ig, immunoglobulin; INF-γ, interferon γ; IL, interleukins; TNF-α, tumor necrosis factor α; SOD, superoxide dismutase; GSH-Px, glutathione peroxidase; CAT, catalase; MDA, malondialdehyde.

The correlative relationships among the FA intake, the levels of FA in serum and milk, the oxidation stability indexes in serum and milk, and the immune function indexes were evaluated in this study (Figure 2). The results showed that C18:0, *cis*-9 C18:1, C18:3 (ALA), and *cis*-9 C18:1 intake was positively correlated with the levels of these FA in serum (p<0.05) (Figure 2A). The levels of *cis*-9 C18:1, ALA, *trans*-11 C18:1, and *cis*-9, *trans*-11 CLA in serum were positively correlated with these FA profiles (p<0.05) and negatively correlated with most SFA in milk (Figure 2B). The levels of *trans*-11 C18:1, ALA, and *cis*-9, *trans*-11 CLA in serum were positively correlated with the concentrations of MDA and IgG (p<0.05) (Figure 2C). The level of ALA was negatively correlated with the concentrations of INF-γ, GSH-Px, and TNF-α in serum (p<0.05). In addition, the level of *cis*-9, *trans*-11 CLA was negatively correlated with the concentrations of GSH-Px, TNF-α, and CAT in serum (p<0.05). The levels of *cis*-9 C18:1, *trans*-11 C18:1, ALA, and *cis*-9, *trans*-11 CLA in milk were negatively correlated with the concentrations of SOD, CAT, and GSH-Px in milk (p<0.05) (Figure 2D). However, the levels of *cis*-9 C18:1 and *cis*-9, *trans*-11 CLA were negatively correlated with MDA concentration in milk. In addition, almost all the levels of SFA in milk were positively correlated with the concentrations of CAT and GSH-Px, whereas they were negatively correlated with the concentration of MDA in milk.

## DISCUSSION

In our previous experiment, we found that 4% of RO or FO supplemented in the diet of dairy cows could increase milk production and enhance the functional FAs content (ALA and CLA) in milk fat [10]. However, any increase in PUFA content would have negative effects on milk oxidation stability [14]. Evidence also suggested that n-3 PUFA affects inflammation through altered gene expressions of inflammatory mediators [6]. This is the first time to our knowledge that the effects of these dietary treatments on the serum FA composition, enzymatic radical scavenging systems both in milk and serum, and the immune responses of dairy cows have been investigated. In this study we also investigated whether the PUFA-rich oils supplemented could impact on dairy cow health. We observed that all the oils used could alter the FA profiles in serum by increasing the levels of *trans*-11 C18:1 (VA) and PUFAs (*cis*-9, *trans*-11 CLA and ALA) without changing the energy balance reflected by the result of energy intake. In addition, the stability of enzymatic radical scavenging systems both in serum and milk were reduced in oil treatment groups. However, the immune function of dairy cows was enhanced by oils treatments.

### Rubber seed oil and flaxseed oil alter fatty acid composition in serum

The addition of RO or FO in the diet of dairy cows increased both CLA and ALA intake (Table 1). A previous study showed that the long-chain fatty acid (LCFA) in blood are mainly due to the uptake from the diet [15]. Previous results from our own research showed that the mean proportion of *cis*-9 C18:1, *cis*-9 C18:2, and ALA in milk were increased in cows fed oils (FO, RFO, or RO), which is in line with the increased concentrations of these FAs in the diet, respectively [10]. In addition, the results show that C18:0, *cis*-9 C18:1, ALA, and *cis*-9 C18:1 intake was positively correlated with the levels of these FA in serum were observed in this experiment (Figure 2A). Thus, the increase in the proportions of *cis*-9 C18:1, *cis*-9 C18:2, and ALA in milk in RO, FO, and RFO groups compared with that in CON group could reflect the addition of these FAs from RO and FO to the diet [10].

In general, FAs in milk are mainly produced from two ways: about 45% of the FAs are short chain and medium chain FAs (C4:0 ~ C14:0, 50% of C16:0), which are synthesised from rumen fermentation products such as acetic acid and β-hydroxybutyric acid, by de novo synthesis in mammary epithelial cells, while the remainder (mainly long-chain FAs, LCFA, all C18 FAs, and 50% of C16:0) are directly absorbed from the blood circulation system [16]. The levels of VA, ALA, *cis*-9, *trans*-11 CLA, and total UFA in serum were significantly increased by oil treatment, and these FAs may form the basis for increasing their content in milk fat as shown in our previous research [10]. Indeed, the profiles of FAs in serum were positively correlated with the profiles of those FAs in milk in the present study (Figure 2A). In addition, the UFA, especially the PUFA, are of vital importance in counteracting the occurrence of spontaneous lipid oxidation [3]. Thus, the increase in the levels of UFA and PUFAs both in serum and milk may also potentially impact on the oxidative stress of cows and milk oxidation stability as discussed below.

### Rubber seed oil and flaxseed oil affect oxidation stability of serum and milk

The antioxidant activity of blood is related to the activity of antioxidant enzymes and the content of lipid peroxide produced by free radical or reactive oxygen species of UFA. Studies have reported that antioxidant enzymes, such as SOD, GSH-Px, and CAT, had the ability to clean up free radicals [17]. The spontaneous oxidation of lipids is a result of many factors, with UFA, especially PUFAs considered as important. Because of the active hydrogen atom in PUFA, the increase in the content of free radicals increases the possibility of spontaneous oxidation [4]. In the present experiment, there was a significant increase in the level of PUFA (CLA and ALA) in serum accompanied by a significant decrease in the activity of GSH-Px and CAT, and an increase in the concentration of lipid peroxide (MDA) in oils groups compared with the CON group. Moreover, the levels of PUFAs (ALA and *cis*-9, *trans*-11 CLA) in serum were positively correlated with the concentrations of MDA and negatively correlated with the concentrations of GSH-Px in serum, which indicates that the increase of PUFA may contribute to decrease the antioxidant capacity and increase the content of lipid peroxidation products. In addition, our previous research showed that the concentration of milk PUFA, especially the ALA, were enhanced by oil treatment [10]. In this experiment, the activity of SOD and CAT in milk of cows receiving oils (RO, FO, or RFO) were significantly lower than that in the CON (p<0.05), which was similar with the results by Liu et al [14] who confirmed that the increase of PUFA in milk could reduce the antioxidant properties of milk and increase the concentration of MDA. Indeed, it was observed in this experiment (Figure 2D) that the levels of PUFA (ALA and *cis*-9, *trans*-11 CLA) in milk are negatively correlated with the concentration of SOD, CAT, and GSH-Px and *cis*-9, *trans*-11 CLA levels and negatively correlated with MDA concentration in milk. Collectively, these results indicate that RO and FO supplementation could reduce the milk oxidation stability and serum antioxidant capacity, which may be associated with the increased levels of PUFA (CLA and ALA) in milk and serum.

### Rubber seed oil and flaxseed oil could enhance host immune function

Ig M is the primary antibody produced in the initial stage of antibody response and IgG plays an important role in the systemic immune response, which has been reported in previous publication [18]. Ig A is the major antibody in mucosal immunity, and its main function is on pathogenic bacteria by intimate cooperation with innate nonspecific defense mechanisms. Previous research showed that supplementing salmon oil to diets with a low ratio of (n-3)/(n-6) PUFA could decrease the immune function [19]. In the present study, the increase in the ratio of (n-3)/(n-6) PUFA in the serum of cows receiving oils (RO, FO, or RFO) compared with CON, could explain the significantly increased concentration of IgG. In addition, both the levels of ALA and *cis*-9, *trans*-11 CLA in serum were positively correlated with the concentrations of IgG in the present study (Figure 2C) indicating that both n-3 and n-6 PUFA may contribute to the immune function, and this requires further investigation. Unlike IgG, the concentrations of IgA and IgM were not influenced by the 4% inclusion of RO or FO supplements. Similarly, IgA and IgM were also not influenced by the post-ruminal infusion of ALA in the serum of the dairy cows [20].

The IFN-γ as an important pro-inflammatory cytokine is produced by T-helper 1 (Th1) lymphocytes that initiate cell-mediated immune responses. Most clinical research in healthy people demonstrates that n-3 PUFA suppresses T cell proliferation, resulting in the depression of the yield capability of T cell-derived cytokines [21,22]. Investigations concerning n-3 FAs showed that dietary n-3 PUFA reduced circulating IFN-γ and IFN-γ mRNA in the spleen using mice as animal models [23]. In the present study it was observed that the oil treatment groups had lower concentrations of IFN-γ in serum and the content of ALA in serum was negatively correlated with the concentrations of INF-γ. These results are inconsistent with previous research showing that duodenal infusion of higher doses of ALA quadratically upregulated IFN-γ levels in the serum of dairy cows, which peaked when the infusion dose was 200 g/d [20]. However, the amount of ALA intake in RO, FO, and RFO groups were 240.1, 490.1, and 370.6 g/d respectively, which exceed the infusion dose 200 g/d and may explain the differences in results between our study and previous studies.

The TNF-α is also an important mediator of the inflam matory response that regulates recruitment and activation of neutrophils. Studies have demonstrated that feeding n-3 PUFA can decrease soluble TNF-α production in rodent macrophages as well as in equine and rats blood monocytes [24], which is similar with our results showing that the concentration of TNF-α in serum was decreased in n-3 PUFA enriched-oil feeding dairy cows. These results are also consistent with Rezamand et al [25] who reported that ALA-enriched diets could reduce the expression of pro-inflammatory markers TNF-α. Indeed, correlation analysis in the present study found that the content of n-3 PUFA (ALA) was negatively correlated with the concentrations of TNF-α in serum. In addition, previous studies showed that diets rich in n-3 PUFA may down-regulate pro-inflammatory markers, possibly through inhibition of transcription factors such as nuclear transcription factor (NF)-κB as TNF-α is an activation factor for NF-κB signalling pathway [24]. Toll-like receptor (TLR4) is an important member of pattern recognition receptors and can activate NF-κB and transcript several proinflammatory genes such as TNF-α [26]. Lee et al [27,28] reported that SFA such as lauric acid stimulated, whereas (n-3) PUFA such as EPA or DHA inhibited, TLR4 activation and the expression of target genes in monocyte/macrophage cells. The markedly increased levels of (n-3) PUFA (DHA and ALA) and decreased levels of SFA in the serum of oil treatment groups could also explain the lower concentration of TNF-α in oil groups. Therefore, previous research in combination with results obtained from the present study leads us to infer that the mechanism by which n-3 FA improves the inflammatory response may be through inhibiting the NF-κB signalling pathway, however, this theory requires further investigation.

The IL-4, and IL-10 are two anti-inflammatory factors, pro duced by Th2 lymphocytes and are predominantly involved in humoral immunity and allergic responses, stimulating antibody production and strong antibody-producing responses. In the present study, both the concentrations of serum IL-4 (p<0.05) and IL-10 (p<0.10) were decreased in FO group compared with RO group, which indicates the anti-inflammatory response was stimulated in cows fed RO. Studies have reported that n-3 PUFA have an anti-inflammatory function because they can influence the production of PGE2, one of the inflammatory factors derived from arachidonic acid [22]. It is well established that PGE2, one of the eicosanoids, is of great importance in regulating the development and role of immune cells and the synthesis of immunoregulatory cytokines [29]. Previous results have demonstrated that administration of linseed oil (n-3 PUFA enrich-oil) significantly lowered the PGE2 synthesising capacity of peripheral blood mononuclear cells in rats [30]. Consistent with those reports, in the present study, concentrations of serum PGE2 significantly decreased in cows receiving FO, when compared with RO.

## CONCLUSION

Diet supplementation with RO and FO could alter serum FA composition by enhancing the content of VA, *cis*-9, *trans*-11 CLA and ALA. In addition, the n-3 FA enriched-oil offered could also enhance the immune function by decreasing pro-inflammatory factors (TNF-α, and IFN-γ). Cows offered RO showed increased levels of anti-inflammatory factors IL-4 and IL-10, however negative effects on milk oxidation stability were observed in the RO group through a reduction in the activity of CAT and SOD. These findings provide a theoretical basis for the application of RO in livestock production.

## Figures and Tables

**Table 1:** Fatty acid (FA) intake of cows fed rubber seed oil and flaxseed oil alone or in combination

Items	Treatment1)				SEM	p-value2)		
	
	CON	RO	FO	RFO		CON vs oil	RO vs FO	RFO vs RO+FO
DMI (kg/d)	20.2	20.5	19.8	19.9	0.26	0.45	0.67	0.37
Energy intake (Mcal/d)	32.5	35.5	34.3	34.4	0.81	0.39	0.68	0.36
Fatty acid intake (g/d)								
C14:0	2.3	3.0	2.5	2.5	0.22	***	*	0.45
C16:0	105.3	161.5	127.6	140.7	1.85	***	**	0.54
C16:1	3.2	4.7	3.1	3.9	0.68	**	**	0.37
C17:0	1.0	1.3	1.3	1.3	0.28	**	0.88	0.87
C18:0	15.0	68.5	37.0	50.6	2.15	***	**	0.55
C18:1 *cis*-9	127.4	284.3	246.1	260.4	4.86	***	*	0.44
C18:2 *cis*-9,12	259.0	526.4	344.4	423.9	5.72	***	***	0.55
C18:3 (ALA)	52.6	240.1	490.1	370.6	4.89	***	**	0.77
C20:0	3.0	5.1	3.4	4.1	0.19	**	**	0.43
C20:1	1.3	2.8	2.5	2.5	0.11	***	0.76	0.45
C22:0	2.6	3.3	3.3	3.3	0.18	*	0.87	0.83
C22:2	2.7	2.9	2.8	2.7	0.11	*	0.44	0.34
Summations (g/d)								
Total C183)	454.0	1,119.3	1,117.6	1,105.4	9.28	***	0.58	0.46
SFA	132.4	248.1	180.2	207.4	4.76	**	**	0.65
UFA	446.2	1,061.1	1,089.0	1,064.0	8.57	***	0.76	0.29
MUFA	132.0	291.7	251.7	266.8	4.75	***	*	0.77
PUFA	314.3	769.4	837.3	797.2	6.74	***	**	0.65

SEM, standard error of least squares means; DMI, dry matter intake; ALA, α-linolenic acid; SFA, saturated fatty acid; UFA, unsaturated fatty acid; MUFA, monounsaturated fatty acid; PUFA, polyunsaturated fatty acid.

1) Cows were fed a basal diet (control; CON) or basal diet supplemented with either 4.0% rubber seed oil (RO), 4.0% flaxseed oil (FO), or 2.0% rubber seed oil+2.0% flaxseed oil (RFO). The CON diet was also used for feeding during the pre-trial period.

2) CON vs oil, CON versus oil (RO, FO, RFO); RO vs FO, RO versus FO; RFO vs RO+FO, RFO versus RO plus FO.

3) Total C18, sum of C18:0, C18:1 *cis*-9, C18:2 *cis*-9, 12, and C18:3 (ALA); SFA, sum of C14:0, C16:0, C17:0, C18:0, C20:0, and C22:0; UFA, total unsaturated FA reported; MUFA, sum of C16:1, C18:1 *cis*-9, and C20:1; PUFA, sum of C18:2 *cis*-9, 12, C18:3 (ALA), and C22:2.

* p<0.05,

** p<0.01,

*** p<0.001.

**Table 2:** Serum fatty acid composition of cows fed rubber seed oil and flaxseed oil alone or in combination

FA (g/100 g of total FA reported)	Treatment1)				SEM	p-value2)		
	
	CON	RO	FO	RFO		CON vs oil	RO vs FO	RFO vs RO+FO
C8:0	0.23	0.28	0.24	0.33	0.02	**	0.09	**
C14:0	0.98	0.76	0.89	0.87	0.05	**	*	0.35
C14:1	0.49	0.38	0.46	0.46	0.02	*	*	0.14
C15:0	0.54	0.37	0.43	0.41	0.02	***	**	0.37
C16:0	8.75	7.81	7.60	7.59	0.16	***	0.31	0.52
C16:1	0.52	0.67	0.69	0.71	0.04	***	0.61	0.55
C17:0	0.72	0.44	0.47	0.44	0.02	***	0.21	0.44
C18:0	12.99	12.79	12.18	11.75	0.28	*	0.11	*
C18:1 t-93)	0.16	0.31	0.28	0.41	0.05	**	0.70	0.06
C18:1 t-11 (VA)	0.41	2.16	2.70	2.89	0.19	***	*	*
C18:1 c-9	9.35	11.28	10.45	10.37	0.56	*	0.26	0.45
C18:2 c-9, 12	48.81	47.50	43.97	45.54	0.81	***	**	0.83
C20:0	1.38	0.70	0.59	0.73	0.08	***	0.32	0.33
C18:3 (ALA)	4.42	6.84	10.74	8.37	0.25	***	***	0.17
C20:1	ND	0.03	0.03	ND	0.00	***	*	***
C18:2 c-9, t-11 CLA	0.01	0.32	0.43	0.46	0.03	***	*	*
C22:0	0.22	0.22	0.26	0.23	0.01	*	***	0.10
C20:3 c-8, 11, 14	3.58	1.37	1.61	1.58	0.16	***	0.27	0.64
C23:0	2.91	2.35	2.17	2.43	0.13	***	0.30	0.24
C22:1 c-13	0.47	052	0.63	0.59	0.02	***	**	0.51
C20:5 (EPA)	0.62	0.74	0.90	0.90	0.05	***	*	0.16
C22:2	0.27	0.23	0.28	0.25	0.01	*	***	0.69
C24:1	0.21	0.16	0.16	0.16	0.01	***	0.65	0.84
C22:4	0.87	0.39	0.40	0.40	0.06	***	0.89	0.99
C22:5	0.90	0.99	0.99	1.09	0.07	0.12	0.99	0.26
Summations								
SFA4)	28.80	25.50	25.20	25.21	0.48	***	0.93	0.45
UFA	71.20	74.43	74.80	74.75	0.48	***	0.91	0.34
MUFA	11.72	15.50	15.40	15.96	0.58	***	0.76	0.44
PUFA	59.48	59.61	59.40	58.77	0.75	0.78	0.96	0.19
(n-3)/(n-6) ratio	0.21	0.22	0.33	0.27	0.01	***	***	0.55

FA, fatty acid; SEM, standard error of least squares means; VA, vaccenic acid; ALA, α-linolenic acid; ND, not detected; CLA, conjugated linoleic acid; EPA, eicosapentaenoic acid; SFA, saturated fatty acid; UFA, unsaturated fatty acid; MUFA, monounsaturated fatty acid; PUFA, polyunsaturated fatty acid.

1) Cows were fed a basal diet (control; CON) or basal diet supplemented with either 4.0% rubber seed oil (RO), 4.0% flaxseed oil (FO), or 2.0% rubber seed oil+2.0% flaxseed oil (RFO). The CON diet was also used for feeding during the pre-trial period.

2) CON vs oil, CON versus oil (RO, FO, RFO); RO vs FO, RO versus FO; RFO vs RO+FO, RFO versus RO plus FO.

3) Expressed as number of carbons: number of double bonds; c = *cis*; t = *trans*.

4) SFA, sum of C8:0, C14:0, C15:0, C16:0, C17:0, C18:0, C20:0, C22:0, and C23:0; UFA, total unsaturated FA reported; MUFA, sum of C14:1, C16:1, C18:1 *trans*-9, C18:1 *trans*-11 (VA), C18:1 *cis*-9, C20:1, C22:1 *cis*-13, and C24:1; PUFA, sum of C18:2 *cis*-9, 12, C18:2 *cis*-9, *trans*-11 (CLA), C18:3 (ALA), C20:3 *cis*-8, 11, 14, C20:5 (EPA), C22:2, C22:4, and C22:5.

* p<0.05,

** p<0.01,

*** p<0.001.

**Table 3:** Enzymatic radical scavenging systems in serum and milk of cows fed rubber seed oil and flaxseed oil alone or in combination

Items	Treatment1)				SEM	p-value2)		
	
	CON	RO	FO	RFO		CON vs oil	RO vs FO	RFO vs RO+FO
Serum								
SOD (U/mL)	42.7	41.8	41.7	47.6	2.68	0.74	0.99	0.10
GSH-Px (U/mL)	122.0	118.7	102.3	104.4	4.23	**	*	0.24
MDA (nmol/mL)	3.8	3.3	4.8	4.6	0.27	0.10	**	0.19
CAT (U/mL)	2.5	2.3	2.0	1.8	0.17	*	0.37	0.27
Milk								
SOD (U/mL)	133.2	127.9	130.6	130.4	1.44	*	0.23	0.56
GSH-Px (U/mL)	72.7	58.8	69.9	56.8	5.28	0.09	0.19	0.28
MDA (nmol/mL)	1.9	3.5	2.1	2.6	0.40	*	0.48	0.12
CAT (U/mL)	5.3	4.0	3.5	3.0	0.31	**	0.46	0.23

SEM, standard error of least squares means; SOD, superoxide dismutase; GSH-Px, glutathione peroxidase; MDA, malondialdehyde; CAT, catalase.

1) Cows were fed a basal diet (control; CON) or basal diet supplemented with either 4.0% rubber seed oil (RO), 4.0% flaxseed oil (FO), or 2.0% rubber seed oil+2.0% flaxseed oil (RFO). The CON diet was also used for feeding during the pre-trial period.

2) CON vs oil, CON versus oil (RO, FO, RFO); RO vs FO, RO versus FO; RFO vs RO+FO, RFO versus RO plus FO.

* p<0.05,

** p<0.01.

**Table 4:** IgA, IgG, IgM, PGE2, and cytokine concentrations in the serum of cows fed rubber seed oil and flaxseed oil alone or in combination

Items	Treatment1)				SEM2)	p-value		
	
	CON	RO	FO	RFO		CON vs oil	RO vs FO	RFO vs RO+FO
IgA (μg/mL)	58.1	73.5	67.4	58.6	1.86	0.44	0.58	0.19
IgG (mg/mL)	30.5	42.5	24.6	23.9	3.15	*	***	0.97
IgM (mg/mL)	4.6	3.9	3.6	4.3	0.63	0.42	0.73	0.50
PGE2 (pg/mL)	12.6	14.6	12.1	12.1	0.55	0.10	**	0.71
Cytokine levels (pg/mL)								
IFN-γ	230.1	213.3	215.2	216.9	1.91	***	0.38	0.42
IL-2	45.3	50.9	45.3	47.9	1.85	0.37	0.29	0.59
IL-4	21.4	22.4	21.4	21.4	0.50	0.36	*	0.08
IL-10	204.1	209.2	201.4	199.9	1.52	0.58	0.07	0.22
TNF-α	67.6	16.3	26.0	23.9	5.79	**	0.89	0.58

SEM, standard error of least squares means; Ig, immunoglobulin; PGE2, prostaglandin 2; INF-γ, interferon γ; IL, interleukins; TNF-α, tumor necrosis factor α.

1) Cows were fed a basal diet (control; CON) or basal diet supplemented with either 4.0% rubber seed oil (RO), 4.0% flaxseed oil (FO), or 2.0% rubber seed oil+2.0% flaxseed oil (RFO). The CON diet was also used for feeding during the pre-trial period.

2) CON vs oil, CON versus oil (RO, FO, RFO); RO vs FO, RO versus FO; RFO vs RO+FO, RFO versus RO plus FO.

* p<0.05,

** p<0.01,

*** p<0.001.
