# Supplementary material for: Rubber seed oil and flaxseed oil supplementation on serum fatty acid profile, oxidation stability of serum and milk, and immune function of dairy cows
Source: Asian-Australas J Anim Sci. 2019 Jan 4;32(9):1363–72. doi: 10.5713/ajas.18.0573 (PMC6722319; doi:10.5713/ajas.18.0573)
Supplement: Supplementary file 1 [file ajas-18-0573-suppl.pdf]

Rubber Seed Oil and Flaxseed Oil Supplementation on Serum Fatty Acid Profile, Oxidation Stability of Serum and Milk, and Immune Function of Dairy Cows

Yu *Pi*[1,2,a], Lu *Ma*[1,a], Hongrong *Wang*[2], Jiaqi *Wang*[1], Jianchu *Xu*[3], and Dengpan *Bu*[1,4,5,*]

[1]State Key Laboratory of Animal Nutrition, Institute of Animal Science, Chinese Academy of Agricultural Sciences, Beijing 100193, China

[2]College of Animal Science and Technology, Yangzhou University, Yangzhou 225009, China

[3]Kunming Institute of Botany, Chinese Academy of Sciences, Kunming 650201, China

[4]Synergetic Innovation Center of Food Safety and Nutrition, Harbin 150030, China

[5]CAAS-ICRAF Joint Laboratory on Agroforestry and Sustainable Animal Husbandry, World Agroforestry Centre, East and Central Asia, Beijing 100193, China

***Corresponding Author:** Dengpan Bu

Tel: +86-010-62813901, **Fax:** +86-010-62813901, **Email:** budengpan@126.com

[a]These authors contributed equally to this work.

Supplementary material

Supplementary Table S1. Ingredient and chemical composition of the experimental diets

Item	Treatment[1)]				SD
	CON	RO	FO	RFO	
Diet ingredient, % of DM					
Alfalfa hay	19.9	19.9	19.9	19.9	1.28
Chinese wildrye	3.8	3.8	3.8	3.8	0.37
Corn silage	24.6	24.6	24.6	24.6	1.92
Corn	17.8	16.2	16.2	16.2	1.44
Flaked corn	6.3	5.7	5.7	5.7	0.62
Soybean meal	11.5	10.5	10.5	10.5	0.98
Soy hulls	2.5	2.3	2.3	2.3	0.30

Distillers dried grains with solubles (DDGS)	3.8	3.5	3.5	3.5	0.40
Double-low rapeseed meal (DLRM)	3.9	3.6	3.6	3.6	0.36
Molasses (30%)	2.5	2.5	2.5	2.5	0.24
Rubber seed oil	-	4.0	-	2.0	0.06
Flaxseed oil	-	-	4.0	2.0	0.06
Sodium bicarbonate	0.5	0.5	0.5	0.5	0.04
Dicalcium phosphate	0.3	0.3	0.3	0.3	0.02
Salt	0.5	0.5	0.5	0.5	0.02
Calcium carbonate	0.9	0.9	0.9	0.9	0.06
Vitamin-mineral premix[2)]	1.2	1.2	1.2	1.2	0.09
Chemical, % of DM (unless otherwise noted)					
DM, %	49.7	50.6	50.6	50.6	0.39
NE_L, Mcal/kg of DM[3)]	1.61	1.73	1.73	1.73	0.06

CP	16.6	16.1	16.1	16.1	0.21
NDF	35.0	34.4	34.4	34.4	0.26
ADF	21.8	21.5	21.5	21.5	0.12
Ca	1.10	1.06	1.06	1.06	0.02
P	0.36	0.35	0.35	0.35	0.01

[1)]Cows were fed a basal diet (control; CON) or basal diet supplemented with either 4.0% rubber seed oil (RO), 4.0% flaxseed oil (FO), or 2.0% rubber seed oil + 2.0% flaxseed oil (RFO). The CON diet was also used for feeding during the pre-trial period.

[2)]Contained (per kg of DM): a minimum of 313,500 IU of vitamin A; 104,500 IU of vitamin D; 5,000 IU of vitamin E; 780 mg of Cu; 780 mg of Fe;780 mg of Mn; 3,900 mg of Zn; 30 mg of Se; 50 mg of I; 65 mg of Co.

[3)]Calculated value (based on China Standard NY/T 34, 2004).
